# Improving NICU staff decision-making with parents in medical rounds: a pilot study of reflective group dialogue intervention

**DOI:** 10.3389/fped.2023.1249345

**Published:** 2023-09-12

**Authors:** Sari Ahlqvist-Björkroth, Ylva Thernström Blomqvist, Jenni Nyberg, Erik Normann, Anna Axelin

**Affiliations:** ^1^ Department of Psychology and Speech-Language Pathology, University of Turku, Turku, Finland; ^2^ Department of Clinical Medicine, University of Turku, Turku, Finland; ^3^ Department of Women’s and Children’s Health, Uppsala University, Uppsala, Sweden; ^4^ Department of Nursing Science, University of Turku, Turku, Finland

**Keywords:** family-centered care, parental involvement, communication, reflective practice, medical round

## Abstract

**Introduction:**

The communication skills of healthcare professionals play a crucial role in successful shared decision-making with parents in neonatal intensive care. Improving communication skills can be achieved through practice and reflection on personal experiences after authentic interaction events with parents. The process of reflection typically involves three phases: description, reflection, and critical reflection. In this study, our aim was to explore the acceptability of the Reflective Group Dialogue intervention and its effectiveness in supporting the reflective process.

**Methods:**

This qualitative pilot study was conducted in the neonatal intensive care unit at Uppsala University Children's Hospital, Sweden. The sample consisted of nine medical rounds with seven families, five neonatologists, seven registered nurses, and five assistant nurses. Purposive sampling was used to collect the data. The intervention comprised four elements: (1) before the intervention, a recorded presentation on shared decision-making was given to the entire unit staff, (2) an observation of a normal medical round discussion with parents, (3) an interview with parents about their experience after the same round, and (4) a reflective discussion with the participating health care professionals after the round. The parent interviews and reflective discussions were audio-recorded and transcribed verbatim. They were analyzed using thematic analysis as a theoretical strategy.

**Results:**

Both parents and staff widely accepted the intervention and found it beneficial. We identified four discussions that remained in the descriptive phase of the reflection process, four that reached the reflective phase, and one that reached the critical reflection phase. The descriptive discussions were characterized by using a single perspective to reflect, often based on personal opinions. The reflective discussions included analyzing interaction sequences from both staff and parent perspectives and were primarily based on actual observations of communication during medical rounds. The critical discussion led to a new awareness of current practices concerning parental involvement in decision-making. These discussions also utilized “what-if” thinking to evaluate potential new practices and their pros and cons.

**Conclusions:**

The intervention seems promising as it was perceived as beneficial by the recipients and facilitated reflection in most cases. However, to enhance the feasibility of the intervention, some improvements are discussed.

## Introduction

Care in neonatal intensive care units (NICU) is undergoing a paradigm change from professional-centered care, which solely focuses on the infant's well-being, to family-centered care (FCC). The inclusion of the family has expanded the focus of care to the well-functioning parent-infant relationship and parenting. According to the FCC model, parents should be actively involved in decision-making about their infant's care (1, 2). Parents have expressed a desire to participate in medical rounds and decisions related to their normal role as parents (3, 4). Parent involvement in decision making in the NICU context has been shown to have an impact on some clinical decisions (5). It has also been shown to increase parents' autonomy, satisfaction with care, feeling heard and respected, and support the development of their parenting and involvement in infant care (6, 7). A meta-analysis of shared decision-making interventions in pediatric contexts showed a decrease in decision-making conflicts and an increase in parental knowledge (8).

The medical rounds represent a traditional institutional practice for information-sharing and decision-making in neonatal intensive care, making it a central practice to consider when improving parental participation in decision-making. Shared decision-making in neonatal care is a collaborative communication process between parents and healthcare professionals, ensuring that decisions align with the patient's values and preferences (2, 9). Communication is both a facilitating and inhibiting factor in successful shared decision-making. Limited information sharing and a lack of explicit discussions and negotiations with parents have been shown to hinder shared responsibility for decision-making, while staff support is crucial in facilitating parents' autonomous decision-making about the daily care of the infant (10). Deficiencies in communication skills among staff can create an implementation gap in FCC in the NICU context (10, 11).

To address this problem, we propose improving individuals' skills in reflecting on their own communication and behavior in decision-making situations with parents. There is evidence, although limited, suggesting that high reflective skills are associated with better communication skills (12). Reflection is a metacognitive process that involves examining one's own experiences afterward and analytically considering them. Through reflection, experiences are mentally reconstructed, reorganized, interpreted, and personal meanings are formed (13). When the reflective process reaches critical reflection, it can lead to a new understanding that supports behavioral changes, such as adopting new communication strategies (14). Reflective thinking is considered a cornerstone of lifelong learning and an essential aspect of professional skills in any work involving direct interaction with patients (15, 16).

Traditionally, the development of reflective thinking in a professional context has been supported using tools focused on personal learning, such as reflective diaries. However, this approach misses the social component of learning and the opportunity for professional reflective dialogue. Recent reviews have reported on the effectiveness of reflective practice interventions that utilize group dialogue within interprofessional healthcare teams, which have improved thinking and facilitated behavioral changes in collaboration and care practices (17–20).

As far as we know there is no reflective practice intervention available for NICU healthcare professionals to enhance their ability to reflect on their communication patterns and the factors influencing communication and shared decision-making in a family-centered way. Therefore, the overall aim of this pilot study was to explore the feasibility of the “Reflective group dialogue” intervention in supporting NICU healthcare teams to involve parents in discussions and decision-making during medical rounds. The specific research objectives were to evaluate the acceptability of the intervention from the perspectives of unit staff and parents and assess its success in facilitating healthcare team members' reflective process on communication and decision-making during medical rounds. The findings from this pilot study will inform the development of the intervention for an evaluation trial to assess its effectiveness.

## Materials and methods

This feasibility study focused on the acceptability of the intervention to the recipients and its success in facilitating reflection. The study was conducted in the NICU at Uppsala University Children's Hospital which is a regional referral NICU (level IIIB). It serves a population of approximately 23,000 births/year from seven county hospitals at distances ranging from 70 to 300 km. The NICU has a long experience in family-centered care by promoting parental presence and participation in the infant's care. The unit has three open-bay intensive care rooms with 12 cots, an adult bed next to each cot, and privacy screens to encourage the presence of parents with their infants. This allows at least one parent to be 24/7 with their infant in the intensive rooms. In addition, the unit has eight single-family rooms, each with a cot. In these rooms, both parents can be with their infants 24/7. All parents at the unit are encouraged to stay with their infant, participate in the infant's care, and participate in the medical rounds (21, 22).

Daily medical rounds were performed in the mornings and involved at least one doctor, one registered nurse, and often one assistant nurse. The parents participated if they were available or awake. The order of progress during the medical round varied slightly, usually depending on the doctor in charge. Generally, the healthcare team would first discuss the infant's current condition and develop a preliminary care plan in a designated room (later on called a doctor's office). Then, the team walked from patient to patient to inform and discuss with available parents. Physical examinations were performed when needed and in the open bay rooms usually before the medical round. Since parents often were present in the unit, in the infant's care room, or on the premises, there were ample opportunities for conversations with doctors outside of the formal medical rounds. A study reported that 89% of mothers and 73% of fathers participated in the medical rounds at the unit (23).

### Participants

The sample consisted of nine medical rounds. A total of seven families attended the nine medical rounds, 7 mothers and 3 fathers. Two mothers attended their infants' medical rounds twice during the study. The inclusion criteria for parents were: (1) their infant was older than 3 days, (2) their infant did not have a life-threatening illness, (3) they could speak and understand Swedish or English, and (4) at least one of the parents was participating in a medical round. Purposive sampling was used to recruit families with infants with different medical conditions and lengths of stay in the NICU.

After a medical round, the healthcare team that took part in the medical round was invited to participate in the reflective dialogue organized by the research team. A neonatologist and a registered nurse participated in all nine discussions, and an assistant nurse also participated in four of the discussions.

### Procedure

The research team approached the families and provided them with an information letter about the study. Written informed consent was obtained from the families who agreed to participate. Following consent from the families, the healthcare team conducting the medical round involving the participating parents was approached and provided with oral and written information. Written informed consent was required from all healthcare team members participating in the infant medical round before data collection.

The data collection was performed for six weekdays between November and December 2018. Out of 150 registered nurses, assistant nurses, and 15 doctors who were approached by email before the intervention, two declined to participate in this intervention pilot. Furthermore, 10 families were approached and seven of them agreed to participate. The background information was collected from the parents and healthcare team members through questionnaires.

### Intervention

The aim of the intervention is to increase the awareness of the healthcare team members of their own attitudes and behavior related to parental participation in communication and decision-making during the medical round. The study received ethical approval from the Swedish Ethical Review Board (Dnr 2018/348). The intervention was developed based on our exploratory study about communication and decision-making during medical rounds (4), an argumentative paper about parents' vulnerability in medical decision-making (4, 24), and our experiences on implementing the Close Collaboration with Parents intervention (25). As a part of the Close Collaboration with the Parents intervention, a systematic structure for the intervention was developed and tested in four Finnish NICUs and one Norwegian NICU between 2016 and 2017.

**Figure 1:**
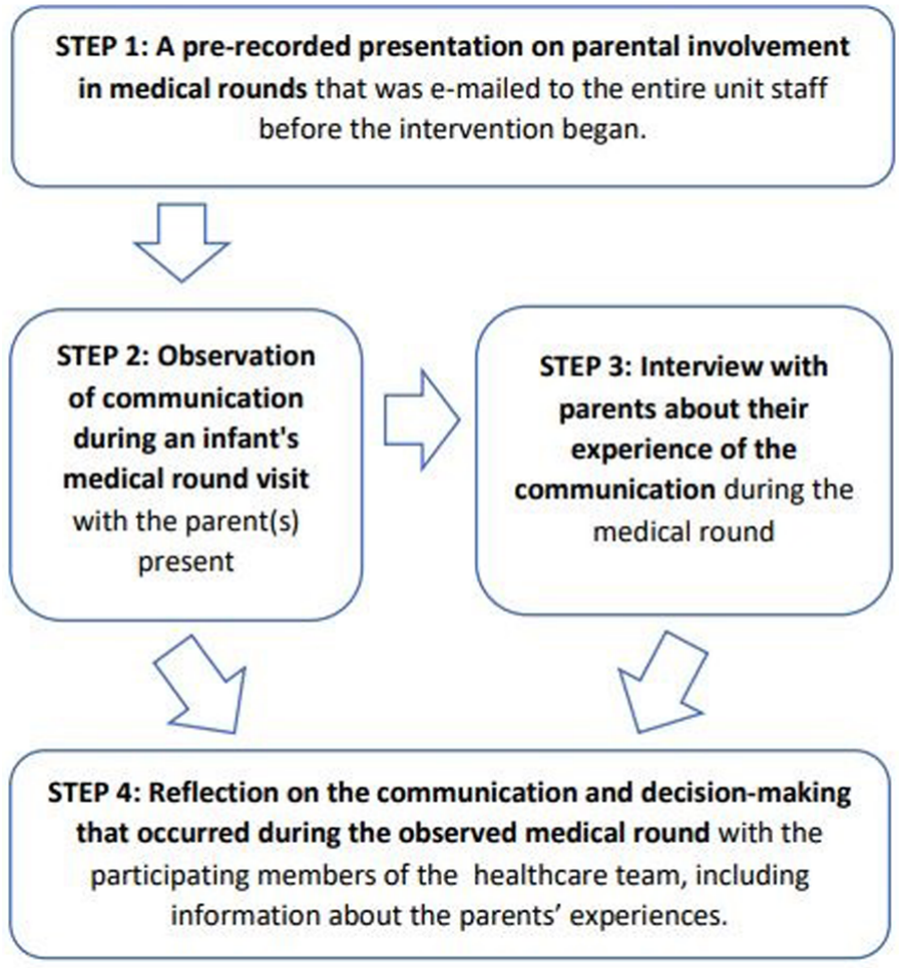
Illustration of the Reflective Group Dialogue intervention and its progression..

The intervention is described in detail according to the Template for Intervention Description and Replication (TIDieR) checklist in Supplementary Appendix S1 (26). In general, the intervention consisted of four elements: (1) a video-recorded presentation on the evidence for parental involvement in medical rounds was e-mailed to all unit staff one month before recruitment, (2) the observation of a medical round that was conducted while the healthcare professionals carried out the round according to their normal practice, (3) an interview of the parents about their experiences of the communication and decision-making during that round, and (4) a reflective discussion with the participating health care professionals regarding communication and decision-making during the medical round (Figure 1).

### Observations of the medical round

Two to three researchers participated as passive observers during the medical rounds. The purpose of this was to both see and hear what happened during the medical round. Memory notes were taken immediately after the round. All researchers who participated as observers during the round also participated in the reflective discussions.

### Parent interviews

Parents were interviewed in their family space/room after the medical round. The semi-structured interview guide included the following themes: parent's thoughts and feelings about (1) the medical round, (2) communication and decision-making during the round, (3) support provided by health care professionals for the participation, and (4) the interventions e.g., observations, providing feedback on healthcare staff, potential participation in reflective discussion At the end of the interview, it was confirmed that parents felt comfortable having their thoughts shared in the reflective discussion. If there were any thoughts that should be kept confidential, they were identified. During the reflective discussion, one of the researchers, at the time, shared insights from the interviews with the parents. The interviews were audio recorded and transcribed verbatim.

### Reflective discussions

The aim of the reflective discussions was to pursue shared awareness and understanding of communication and decision-making between healthcare professionals and parents during medical rounds, using reflective questions (Supplementary Appendix S2). This conversation should work as an enabler for critical reflective thinking, allowing each participant's emotions and ideas to be expressed, which may lead to new understanding and thereby changes in practices. A researcher also incorporated insights from parents' perspectives gained through the interviews into the discussion (Supplementary Appendix S3). The reflective discussions were held in a separate room near the NICU and they lasted from 45 to 60 min. At the end of the discussions, feedback about the intervention was requested. The reflective discussions were recorded and transcribed verbatim.

Three researchers facilitated the reflective discussions, one at a time. One was a psychologist with psychotherapeutic training that included the use of reflection in the therapeutic context. She and another researcher on this team had created the facilitation structure for the reflective discussion and had tested and modified it in four different NICUs prior to this pilot. The third researcher learned facilitation by observing and later facilitating the discussions conducted toward the end of the study and receiving feedback from others.

### Analysis strategy

The theoretical thematic analysis was used as an analysis strategy (27). The phases of the reflection process in each discussion were examined using the set of theory-based criteria that combined Koole's cyclic reflection process (14), Kember's levels of reflective thinking (28), and psychotherapeutic understanding of the reflection concept which added emotions and mentalization in the reflection process (16). The criteria for three phases of the reflection process (1) description, (2) reflection, and (3) critical reflection are described in Table 1.

**Table 1:** The criteria for the phases of reflection used in the analysis.

Phases of reflection process	Applied criteria for the reflective group discussions	Structure of the descriptions of the cases
1. Description	Reviewing the experience of the medical round situation, the behavior of the family and healthcare members Introspection: Becoming aware of one's own thoughts, feelings, reactions, and views of contextual factors Applying previous knowledge	Starting point: Introspection focusing on participants’ own thoughts and opinions. Type of used description: Descriptions about the purpose of the medical round. Used previous knowledge: Reasoning the difficulty to involve the parents in the medical rounds or in the decision-making. Response to critical observations in the group: Did not trigger any reflection.
2. Reflection	Analytic exploration of the medical round experience from the point of view of interaction and decision-making; by reflective questions and searching for answers to the questions. Forming an interpretation of the events that happened during the medical round; validating, complementing, or changing the interpretation. Metalizing reflection: Mentalizing one's own and others’ thoughts, feelings, behavior, and the interaction they form.	Starting point: Recalling the events from the observed medical rounds. Type of used description: Description of interaction turns between staff and parents. Used analytic exploration: The participants explored both their own and parents’ behavior. Response to critical observations in the group: The parents’ comments triggered some critical inquiries. Forming an interpretation: The interpretations of the interaction events.
3. Critical reflection	Becoming aware of the motives behind communication behavior, evaluating critically the current practices, and thinking about medical rounds or decision-making from the perspective of involvement of families. Making conclusions and action plans Verbalization of the change in the current viewpoint and reflection on the potential impact on behavior or practices.	Starting point: Recalling the events from the observed medical rounds. Type of used description: Description of interaction turns between staff and parents. Used analytic exploration: Analytic exploration of the current medical round practice from the perspective of parental involvement. Response to critical observations in the group: A comment from a new staff member triggered a critical exploration of the current medical round practice from the perspective of parental involvement. Forming an interpretation: Interpretations of the involvement of the family to the decision-making. Making conclusions: About what needs to be done to improve the practices. Reflection on the potential impact: Exploration of the impact of proposed practice change.

First, the transcribed reflective discussions were coded according to the criteria representing different reflection phases. This allowed us to evaluate to which phase the reflection process progressed during each group discussion. In the next phase, the codes under each phase were grouped into the sub-themes which formed the structure of the description for each phase. The structure of each phase of the reflection process was different. However, in all three, we described first the starting point of the discussion and what kind of descriptions were used at that specific phase. The discussions in the reflection and critical reflection phases continued further to the analytic exploration, responses to new or critical observations, and forming interpretations. Only in the critical reflection phase, we were able to identify plans for new practices and reflection on their potential impacts (Table 1). The description of the sub-themes was embodied in the content of the included reflective group discussion in each phase. From the parent interviews only, the comments related to the acceptability of the intervention were coded inductively. The initial analysis was done by (JN) and confirmed by (SAB). The disagreements were given to another researcher for review (AA). The final classification of the discussions to different phases of the reflective process is in accordance with the consensus between two researchers (SAB & AA). The analyses were conducted with the help of NVivo 12 software.

## Results

Altogether seven mothers and three fathers of the infants participated in the nine medical rounds and interviews after the round. The average age for the mothers was 27.8 years and 35.1 years for the fathers. All the infants were singletons and their average gestational age at birth was 29 weeks. The healthcare teams that participated in the intervention sessions consisted of five neonatologists, seven registered nurses, and five assistant nurses. Three of the neonatologists were men and two were women with an average age of 46 years. All the registered nurses and assistant nurses were women. The average age of the registered nurses was 42 years and assistant nurses were 40 years. Of the participating healthcare team members, 83% worked full-time in the unit.

### The acceptability of the intervention

All parents who participated in the intervention agreed with the researchers' presence at their infant's medical rounds. They expressed that they were used to many people attending medical rounds. Parents expressed that they were able to act and discuss normally during the rounds. All parents described it as a positive experience to be heard and to give feedback about the medical round. They wanted without hesitation to share their experiences with the healthcare team.

*A parent: You are quite used to it here, it is often students, and there are often many people, so you are quite used to it. (Parental interview case 7)*

Furthermore, parents could consider participating in a reflective discussion with the healthcare team after the round. They felt that it would be an important opportunity to develop care and provide constructive critique to the unit staff. However, one parent also brought up that it could be problematic to provide critique to the healthcare staff although it is the parent's right and responsibility.

*A parent: Or maybe like ask if the parents want to. Maybe ask if you want to explain more, it might, as a parent when you are in this situation where you don't want the people that are to save your kid's life to feel bad towards you if you have given some kind of critique, and it might be put in another way if…ethically I think it would be a good idea that the parent can explain because it's not really in your interest, it is in the parents interest that the doctor and you yourself have a good…(Parental interview case 2)*

Almost all approached healthcare team members of the unit were willing to participate in the intervention pilot. However, none of those who participated had reviewed the video material provided prior to the intervention. All reflective discussions were carried out during the day the round observation was conducted, even though finding the dedicated time for the discussions within the unit's busy daily schedules was a challenge. The challenges were: (1) difficulties in scheduling a common time for all healthcare team members who participated in the observed round in three cases, (2) interruptions during the reflective discussions in one case, and (3) delay in attending to or leaving in the middle of the discussion in three cases.

All the healthcare teams that participated in the reflective dialogue sessions reported that the discussions were somewhat beneficial. The team members appreciated the reflective discussions because they offered a ground for multi-professional reflections and reflections on situations other than extremely acute situations. They also reported that the discussions facilitated the emergence of new ideas, learning, and the identification of needed improvement needs. The team members valued the parents' feedback, which especially stimulate new ideas or validate the current practices. They mentioned that a busy day in the unit and a lack of skills or courage to reflect were the main hindrances to success in reflective discussions. They also assumed that the timing of the discussion immediately after the observed medical round and keeping it brief would facilitate the success of reflection.

### The success of the intervention to support healthcare team members' reflective process

In the deductive analysis, we found that out of nine reflective discussions, five included some form of reflection. Based on the level of reflection expressed in the content of discussions three groups were identified: (1) descriptive discussions, (2) reflective discussions, and (3) discussions with critical reflection.

## Descriptive discussions

These discussions mainly focused on events during the medical round, often starting with introspection or descriptions of practices. Participating healthcare team members applied some previous knowledge to understand parents' participation in the round, but actual events were rarely recalled.

Introspection was used when commenting on the medical rounds, where participants examined their own thoughts and feelings. For example, they evaluated their satisfaction with the round performance. They experienced the rounds as easy, mainly because they were familiar with the parents. They also described familiarity brought them security and made communication easy with parents. Introspection was also seen in comments where the participants expressed their personal preferences regarding the medical round practices.

*Healthcare team member 3: I personally do not like it, but there are two different types of discussions, a round without the parents and then talking with them later on. Personally, I do not like rounds without parents. (Reflective discussion, case 7)*

In addition, the descriptive comments used focused mostly on the purpose and structure of the round. For the healthcare staff, the main purpose of the round was to gather information and make plans. They also emphasized the importance of parental participation, both intellectually and in general. They emphasized that the round was for the parents and that the parents knew their children best. They described that a parent who is present in the unit a lot can be a source of information for decision-making, although the doctor has the actual competence to make the decisions. Teams also described how communication during rounds becomes a continuum when parents are present daily. Contradictorily, they also concluded that it did not make a difference whether the parents were present or not because the decisions could be made without them and the parents received the information afterward. No one recognized this inconsistency between the comments. Overall, these descriptions were not directly related to the observed medical rounds.

In these discussions, the previous knowledge was applied to reason the difficulty of involving parents in the medical rounds or decision-making. The reasons were related to the nature of the unit, professionals' own needs, and assumptions about the parents. As the unit was a teaching hospital, it was seen as hindered to parental participation due to potentially awkward situations arising from teaching, such as parents overhearing what they might perceive as “stupid questions”. Experienced team members also expressed a need to discuss and consider decisions among themselves before involving parents. The computer was recognized as an important tool for decision-making but was also seen as potentially disrupting contact with parents, creating a dilemma between bedside decision-making and decision-making in a doctor's office. Furthermore, team members made assumptions about parents that hindered their invitation to participate, believing that parental involvement would be too stressful for them.

*Healthcare team member 1: You know, that the mother knows her child the best, that you take it into account, and we did. It (the round) is for them*.

*Healthcare team member 2: I totally agree but you cannot put too much pressure on her. They've been here for a long time, and it's starting to get stressful for her, I have a feeling. She is also quite young. And we tend to forget that it's the first child and she has no relative to backup. I wouldn't put pressure on her about participating in the rounds. (Reflective discussion, case 3)*

Some team members would have liked to involve the parents more in decision-making than the current practice enabled. However, these individual critical voices did not trigger any further discussions in these discussions. Instead, the other team members wanted to convince others that there were no reasons to be critical, asserting that “We did everything just fine”. Although this comment was made with good intentions, it suppressed the opportunity for reflective exploration.

Overall, these discussions had a strong tendency to switch over to a general discussion about the medical rounds from the exploration of the observed medical round situations. Only one actual event was described in detail during these discussions. The descriptions were also mainly focused on parents' behavior or participants' own behavior but not on parent-staff communication or interaction. Personal preferences regarding practices were often emphasized in the comments. Overall, participating teams expressed satisfaction with the current practices related to medical rounds.

## Reflective discussions

In these cases, the healthcare teams' discussions were primarily grounded in the actual events that occurred during the medical rounds. For instance, a team described their communication with a mother who was worried about her infant. They became aware that the mother's worry influenced how they communicated with her. They explored analytically their own behavior, emphasizing the importance of attentive listening and providing reassurance throughout the discussion. The team felt that this approach met the mother's needs. Their interpretation was that they consciously reduced the amount of detailed information shared to help regulate her anxiety. A participant also shared an observation of a healthcare team member's words bringing immediate relief to a mother.

*Healthcare team member 4: “I think you said exactly that, it looks good….You see that she is relieved and exhales, the same thing when you confirm that the baby has pooped, that's great, then you see that she exhales, you see in her body language”. (Reflective discussion, case 2)*

Another team discussed how their prior collaboration with a parent influenced their trust in the parent's knowledge about their infant during the medical rounds. They recalled offering the parent two options for increasing milk amounts and allowing the parent to make the final decision. They interpreted that familiarity with the parent facilitated sharing observations and involvement in decision-making. They concluded that it was easier to interpret a parent's behavior when they knew the parent. They also concluded that knowing a parent's educational level could be meaningful in adjusting communication.

The descriptions in these discussions often centered around parents. In the discussion of a team, the critical inquiry stemmed from surprising information shared by a parent that could have been useful in decision-making but was not disclosed to the healthcare team. The team reconstructed the interaction during the decision-making situation in detail, leading to the interpretation that the healthcare team's behavior, such as turning toward each other, asking questions to one another, and limiting eye contact, caused the parent to withdraw. The team become aware that positioning and eye contact played a crucial role in parent and team member participation. As an improved practice, they proposed standardized positions in a circle during medical rounds.

*Healthcare team member 1:… But the one who stands opposite is asked the question more easily or more often, I feel. I think that the mother was standing opposite me and I talked to her so when I heard this question I turned to you, then I had my back towards the mother …*

*Healthcare team member 3: …. I do not know, but it may be something like that because it may be clear that I was turning away from the mother. Then she feels excluded from the discussion, she needs to be very persistent to get into the discussion again, not just eye contact, maybe with physical contact or using a loud voice*.

*Healthcare team member 1: Should we position ourselves in a standardized way from the beginning because it is so important for communication? (Reflective discussion, case 2)*

Another team interpreted a parent's behavior during a round as a need for control. They try to understand parents' behavior by mentalizing how it may feel if one suddenly loses the sense of control. Some participants were also mentalizing how their communication and behavior during the medical round might make parents feel.

These discussions included genuine, varied, and detailed observations of the actual medical round situations. Descriptions focused on activities during the round and the interaction between the healthcare team and parents. The participants reconstructed collaboratively the staff-parent communication or decision-making situations, resulting in a comprehensive understanding of the episode. The families' perspectives were also acknowledged throughout the discussions, and empathy was expressed toward them. Team members aimed to understand their own and parents' feelings and behaviors through mentalization. Feedback from parents played a crucial role in triggering critical inquiry in one case.

## Discussions with critical reflection

In this case, the discussion began by recalling the interaction between the healthcare team and parents during the observed round. The descriptions mainly focused on the roles of the participants in the discussion. The team noted that during the medical round, today's discussion was mainly for the parent. The nurse's role was described as a facilitator, allowing parents to voice their observations and concerns more openly. They also recognized that at a certain point in the round, the roles of active participants changed. This occurred when a nurse posed an important question about necessary blood tests, leading to a discussion between the nurse and the doctor. These descriptions made the team become aware that everyone contributed something valuable to the discussion, and this active involvement allowed decisions to be made collaboratively.

A critical reflection cycle was initiated by a team member who was new in the unit. This member realized that the current structure of the medical rounds did not support the inclusion of parents in the rounds in intensive care rooms. The other group members agreed with this observation. They also became aware of the paradox that while no one opposed parents' participation, they were still not actively inviting them. They recalled a previous unsuccessful attempt to involve parents in the medical rounds in the intensive care rooms. However, they also identified some benefits, such as more dynamic dialogue in rounds where parents were involved and the ability of parents to raise meaningful issues that the healthcare team had not considered. After reflecting on the previous effort, the group proceeded to explore what would change if they actively involved parents in the rounds in the intensive care rooms. They concluded that it might save time.

*Healthcare team member 1: But he is right in saying that we do not actively invite the parents to the round. Nobody thinks it's weird if they are involved but we do not actively invite them. You can see that as a disadvantage*.

*Healthcare team member 2: We do not tell them that our rounds start at half-past eight and ask if they want to join, that they are welcome. (Reflective discussion, case 4)*

In this case, the team also analytically explored the meaning of parents' participation in the medical rounds. They pondered whether the purpose of parents’ participation was to involve them in decision-making or simply to provide an opportunity for parents to meet with a doctor. Meeting a doctor could happen at any time, but changes need to be made if the true purpose was to involve parents in decision-making.

Based on the team's newfound understanding, they began to explore potential practice changes. They focused on finding the appropriate setting for sensitive discussions with parents about infant care, particularly regarding critical care decisions. The main discussion revolved around whether decision-making should occur outside the intensive care room or in a “doctor's office”, and whether parents could participate in decision-making if critical decisions were made in the doctor's office. A participant recalled instances where parents had requested to be present in the doctor's office for decision-making. However, the group interpreted this as only possible for strong-willed parents. Eventually, they ended up evaluating the potential new practice of involving parents in decision-making when it takes place in the doctor's office.

*Healthcare team member 1: Could we take the parent in there (to the office)?*

*Healthcare team member 2: Absolutely, we could do that*

*Healthcare team member 1: There may be patient papers and patient information on the computer screen*.

*Healthcare team member 2: Yes, there are things that we need to think about. I do not really think that anyone is negative about having parents, it is not a problem. It's more of a logistical issue*.

*Healthcare team member 3: Logistical and confidentiality issues. (Reflective discussion, case 4)*

The team demonstrated the ability to critically analyze their current practices regarding parents' involvement in medical rounds. They became aware that decisions in the intensive care room were often made without parents because they were not invited to the medical rounds. The group engaged in analytical exploration using “what-if” thinking and evaluated potential new practices, considering their benefits and drawbacks. Overall, the topics were examined from various perspectives. Interestingly, while the group reached a level of critical reflection, the discussion did not involve much recollection of events from the observed round.

## Discussion

The aim of this study was to evaluate the acceptability of the Reflective Group Dialogue intervention and its success in supporting NICU healthcare team members' reflective process regarding their communication with parents during a medical round. The intervention was well accepted by both the parents and the healthcare team members, who found it beneficial. However, there were some challenges in delivering the intervention practically. The intervention facilitated a reflective process in 5 out of 9 discussions after the medical round. We identified four discussions that remained at the descriptive level, four that reached the reflective level, and one that reached the critical reflection level.

The approached healthcare team members and parents were mostly willing to participate in the intervention. The parents would have been even willing to participate in a reflective group discussion with staff because they valued being heard. The main challenge was to integrate the intervention into the daily routines of the unit. Challenges included finding a convenient time for all team members and ensuring proximity to the observed medical round which would have allowed for fresh recollections of events. The healthcare team members also recognized the advantage of reflecting on staff-parent communication after everyday medical round situations. This differs from the typical use of debriefing methods, which are often employed in the context of simulation teaching or after adverse or critical situations. Furthermore, healthcare team members expressed that feedback from the parents' interviews was valuable to them.

In the four cases where the intervention did not succeed in facilitating reflection, a majority of the healthcare team members expressed strong satisfaction with the round events, which may have diminished their motivation for reflection. The observed medical rounds may have been too routine-like, lacking the elements that would trigger the reflective process. Humans are naturally inclined to respond to changes and variations in the environment, paying more attention to unexpected events, while routines or typical events do not rise our interest (16). Furthermore, the lack of motivation may be due to a lack of prior experience in reflection and an understanding of its benefits. Therefore, providing an introductory lecture or material prior to the intervention to frame the value and intended outcomes of reflective discussions would have been important (20). In these discussions, only a few critical comments were made, and they were not explored further. This may be due to participants not feeling safe enough to explore the critical comments (16). An interesting inconsistency was also identified in these discussions: healthcare team members expressed appreciation for parents' involvement but perceived their actual participation in the medical rounds as insignificant. However, neither the participating team members nor the interventionist, as the discussion facilitator, pointed out this inconsistency, which could have prompted reflection. Furthermore, in the descriptive discussions, personal opinions were emphasized, which may have hindered reflection from the perspective of another person (29). This is typical for introspection, where reflection focuses inward, for example, examining one's thoughts and feelings (16). In these cases, facilitators could have encouraged group members to consider the situation from a parent's perspective. The descriptive discussions should not be simply dismissed as non-reflective, as some attempts to understand and reflect were present (28). However, the differences were not clear enough to form a distinct fourth reflection phase between description and reflection.

The intervention primarily facilitated reflective discussions, which is in line with previous findings that achieving lower levels of reflection is more common than achieving higher levels (17, 30). The reflective discussions were most strongly grounded in the observed situations and staff-parent interactions. This is also consistent with earlier research indicating that using clinical situations is a key element of effective reflective practice interventions (20). In these discussions, participating healthcare team members reconstructed interaction chains and engaged in analytical and collaborative exploration. They all appeared motivated to have reflective dialogues. In contrast to the non-reflective discussions, the observed rounds included factors that may have increased participants' motivation for reflection. The observed situations evoked emotions in the team members, and comments from parents' interviews introduced surprising perspectives that fostered the reflective process. The unexpected information provided alternative viewpoints or explanations for the events during the observed situations. Considering alternatives is a central aspect of the reflective process (16). Participants also spontaneously engaged in mentalization in these discussions by considering the situation from the parents' perspective more often than in non-reflective discussions. They also expressed empathetic understanding towards parents. However, the reflective process often stopped at empathetic understanding and did not progress to critical reflection on how these observations could be used to improve parents' participation in the future. Critical reflection could have involved questioning one's own actions, behaviors, or interpretations and exploring alternative options. Perhaps facilitators could have more actively suggested these reflective actions to the groups.

Critical reflection is often described as a change of deeply held or fundamental assumptions or preceding transformative learning (31). The deeply held assumptions are typically something that a person does not question easily rather one selects the observations from the environment that supports the assumption or makes them fit with the assumption. In the discussion that reached critical reflection, the starting point was a questioning of current practices by a new team member who was probably still able to observe the practices with the eyes of an outsider. In this discussion, the transformative idea of the group was that parents' possibility to meet a doctor is different from being involved in decision-making. This may seem a small change of assumption but its impact on practice is big. Instead of being satisfied with parents' possibility to meet a unit doctor anytime and keeping parents well informed the group realized that they need to change something if they want to involve parents in everyday decision-making. Furthermore, the theoretical idea that the unearthed assumptions often relates to power relationship is also embodied in this case (16, 31). The participating team members realize that it is in their hands to support making the change. Critical reflection can be described as deconstruction that then can be followed by reconstruction (31). In this case, the group started to reconstruct by proposing and evaluating potential new practices. The next step would have been to follow if this discussion led to some behavioral changes but that was not the aim of our study.

The credibility of our findings is supported by the earlier literature on the phases of the reflection process and systematic data analysis conducted by three researchers. However, our description of critical reflection is based on only one reflective group discussion. In the future, it is important to further study the common patterns of this kind of reflective process. Another limitation to consider is the varying expertise levels of the facilitators of the reflective group discussions, which may have influenced the course of the discussions and potentially diminished the dependability of our data collection process. However, in many NICUs, it is still common practice for parents not to be allowed to participate in medical rounds. In such contexts, the acceptability of our intervention would be questionable. The whole research team engaged in regular reflective discussions on our impact on the study findings during the data collection and analysis process. Although the research team spent time in the study unit and two of its members were unit staff members, the participants, and researchers might not have established a trusting enough relationship, which is required for the critical reflection process.

## Conclusion and implications

The Reflective Group Dialogue intervention seems promising, as recipients found it useful and it facilitated reflection in most cases. The intervention facilitated reflection in cases where healthcare members reconstructed the actual interaction sequences themselves and parents, and when they were ready to explore the critical comments, several perspectives, and alternatives that emerged during the intervention session. These prerequisites appear to be necessary for the progression of the reflection process.

However, there are areas for improvement to make the intervention more feasible. The main challenge was finding a suitable time for the intervention session within a regular working day in the NICU. Allocating dedicated time resources for the reflective group dialogue session could facilitate the mindful curiosity required for observing interaction sequences and engaging in self-reflection. Prior to the intervention, an introductory session or materials could be provided to establish the theoretical background, value, and intended outcomes of reflective group dialogue. Repeating the intervention session may also support the progression of the reflective process. In addition, the use of video replay of the medical round communication could enhance recall of staff-parent interaction events and facilitate reflection. Anyway, introducing a non-judgmental approach to reflective practice prior to the intervention may be important to create psychological safety for the group dialogue sessions. We highlighted the non-judgmental approach only at the beginning of reflective dialogue sessions. Additionally, encouraging the teams to select medical round situations that include elements (such as challenging or emotional discussions with parents) that potentially trigger the motivation to reflect could be beneficial. Thus, the selection of a challenging case requires trust in the discussion facilitator and team members. Parents' feedback on communication during medical rounds, as well as the viewpoints of new team members regarding unit practices, can bring new perspectives and thus facilitate the reflective process.

Our intervention could provide a method for NICU healthcare teams to actively explore current communication practices and implement the FFC approach in their everyday communication. Furthermore, it may provide an active way to involve parents in co-creating the communication culture within a unit.

## Data Availability

The datasets presented in this article are not readily available because The consent was obtain only for this study. Requests to access the datasets should be directed to sarahl@utu.fi.

## References

[1] Gooding JS Cooper LG Blaine AI Franck LS Howse JL Berns SD. Family support and family-centered care in the neonatal intensive care unit: origins, advances, impact. Semin Perinatol. (2011) 35(1):20–8. 10.1053/J.SEMPERI.2010.10.004 21255703

[2] Hoffmann T Bakhit M Michaleff Z. Shared decision making and physical therapy: what, when, how, and why? Braz J Phys Ther. (2022) 26(1):100382. 10.1016/J.BJPT.2021.100382 35063699 PMC8784295

[3] Abdel-Latif ME Boswell D Broom M Smith J Davis D. Parental presence on neonatal intensive care unit clinical bedside rounds: randomised trial and focus group discussion. Arch Dis Child Fetal Neonatal Ed. (2015) 100(3):F203–9. 10.1136/archdischild-2014-306724 25711125 PMC4413798

[4] Axelin A Outinen J Lainema K Lehtonen L Franck LS. Neonatologists can impede or support parents’ participation in decision-making during medical rounds in neonatal intensive care units. Acta Paediatr. (2018) 107(12):2100–8. 10.1111/apa.14386 29723925

[5] Bailey SM Hendricks-Muñoz KD Mally P. Parental influence on clinical management during neonatal intensive care: a survey of US neonatologists. J Matern Fetal Neonatal Med. (2013) 26(12):1239–44. 10.3109/14767058.2013.776531 23414460

[6] Treherne SC Feeley N Charbonneau L Axelin A. Parents’ perspectives of closeness and separation with their preterm infants in the NICU. J Obstet Gynecol Neonatal Nurs. (2017) 46(5):737–47. 10.1016/j.jogn.2017.07.005 28802557

[7] Voos KC Ross G Ward MJ Yohay A-L Osorio SN Perlman JM. Effects of implementing family-centered rounds (FCRs) in a neonatal intensive care unit (NICU). J Matern Fetal Neonatal Med. (2011) 24(11):1403–6. 10.3109/14767058.2011.596960 21801140

[8] Wyatt KD List B Brinkman WB Prutsky Lopez G Asi N Erwin P Shared decision making in pediatrics: a systematic review and meta-analysis. Acad Pediatr. (2015) 15(6):573–83. 10.1016/j.acap.2015.03.011 25983006

[9] Entwistle VA Watt IS. Patient involvement in treatment decision-making: the case for a broader conceptual framework. Patient Educ Couns. (2006) 63(3):268–78. 10.1016/j.pec.2006.05.002 16875797

[10] Pellikka H-K Axelin A Sankilampi U Kangasniemi M. Shared responsibility for decision-making in NICU: a scoping review. Nurs Ethics. (2023):096973302211349. 10.1177/09697330221134948 PMC10185855 36688269

[11] Wreesmann WW Lorié ES van Veenendaal NR van Kempen AAMW Ket JCF Labrie NHM. The functions of adequate communication in the neonatal care unit: a systematic review and meta-synthesis of qualitative research. Patient Educ Couns. (2021) 104(7):1505–17. 10.1016/J.PEC.2020.11.029 33341329

[12] Karnieli-Miller O Michael K Gothelf AB Palombo M Meitar D. The associations between reflective ability and communication skills among medical students. Patient Educ Couns. (2021) 104(1):92–8. 10.1016/j.pec.2020.06.028 32624329

[13] Kinsella EA. The art of reflective practice in health and social care: reflections on the legacy of Donald Schön. Reflective Pract. (2010) 11(4):565–75. 10.1080/14623943.2010.506260

[14] Koole S Dornan T Aper L Scherpbier A Valcke M Cohen-Schotanus J Factors confounding the assessment of reflection: a critical review. BMC Med Educ. (2011) 11(1):104. 10.1186/1472-6920-11-104 22204704 PMC3268719

[15] Guest CB Regehr G Tiberius RG. The life long challenge of expertise. Med Educ. (2008) 35(1):78–81. 10.1111/j.1365-2923.2001.00831.x 11123600

[16] Stedmon J Dallos R. Reflective practice in psychotherapy and counselings. Maidenhead: Mc Graw Hill Open University Press (2009).

[17] Mann K Gordon J MacLeod A. Reflection and reflective practice in health professions education: a systematic review. Adv Health Sci Educ. (2009) 14(4):595–621. 10.1007/s10459-007-9090-2 18034364

[18] Nancarrow SA Smith T Ariss S Enderby PM. Qualitative evaluation of the implementation of the interdisciplinary management tool: a reflective tool to enhance interdisciplinary teamwork using structured, facilitated action research for implementation. Health Soc Care Community. (2015) 23(4):437–48. 10.1111/hsc.12173 25522769

[19] Petersson P Springett J Blomqvist K. Telling stories from everyday practice, an opportunity to see a bigger picture: a participatory action research project about developing discharge planning. Health Soc Care Community. (2009) 17(6):548–56. 10.1111/j.1365-2524.2009.00854.x 19840129

[20] Richard A Gagnon M Careau E. Using reflective practice in interprofessional education and practice: a realist review of its characteristics and effectiveness. J Interprof Care. (2019) 33(5):424–36. 10.1080/13561820.2018.1551867 30513235

[21] Raiskila S Lehtonen L Tandberg BS Normann E Ewald U Caballero S Parent and nurse perceptions on the quality of family-centred care in 11 European NICUs. Aust Crit Care. (2016) 29(4):201–9. 10.1016/J.AUCC.2016.09.003 27720034

[22] Raiskila S Axelin A Toome L Caballero S Tandberg BS Montirosso R Parents’ presence and parent-infant closeness in 11 neonatal intensive care units in six European countries vary between and within the countries. Acta Paediatr. (2017) 106(6):878–88. 10.1111/apa.13798 28235152 PMC5434801

[23] Aija A Toome L Axelin A Raiskila S Lehtonen L. Parents’ presence and participation in medical rounds in 11 European neonatal units. Early Hum Dev. (2019) 130:10–6. 10.1016/j.earlhumdev.2019.01.003 30639968

[24] Uusitalo S Axelin A. Opioid-dependent mothers in medical decision making about their infants’ treatment: who is vulnerable and why? Les Ateliers de L’éthique. (2018) 12(2–3):221–42. 10.7202/1051283ar

[25] Ahlqvist-Björkroth S Boukydis Z Axelin AM Lehtonen L. Close collaboration with Parents™ intervention to improve parents’ psychological well-being and child development: description of the intervention and study protocol. Behav Brain Res. (2017) 325:303–10. 10.1016/j.bbr.2016.10.020 27743940

[26] Hoffmann TC Glasziou PP Boutron I Milne R Perera R Moher D Better reporting of interventions: template for intervention description and replication (TIDieR) checklist and guide. Br Med J. (2014) 348(mar07 3):g1687. 10.1136/bmj.g1687 24609605

[27] Braun V Clarke V. Using thematic analysis in psychology. Qual Res Psychol. (2006) 3(2):77–101. 10.1191/1478088706qp063oa

[28] Kember D McKay J Sinclair K Wong FKY. A four-category scheme for coding and assessing the level of reflection in written work. Assess Eval High Educ. (2008) 33(4):369–79. 10.1080/02602930701293355

[29] Franco RS Franco CAGdS Severo M Ferreira MA Karnieli-Miller O. Reflective writing in the teaching of communication skills for medical students—a systematic review. Patient Educ Couns. (2022) 105(7):1842–51. 10.1016/j.pec.2022.01.003 35063310

[30] Hulsman RL Harmsen AB Fabriek M. Reflective teaching of medical communication skills with DiViDU: assessing the level of student reflection on recorded consultations with simulated patients. Patient Educ Couns. (2009) 74(2):142–9. 10.1016/j.pec.2008.10.009 19062232

[31] Brock SE. Measuring the importance of precursor steps to transformative learning. Adult Educ Q. (2010) 60(2):122–42. 10.1177/0741713609333084

