# Supplementary material for: Improving NICU staff decision-making with parents in medical rounds: a pilot study of reflective group dialogue intervention
Source: Front Pediatr. 2023 Sep 12;11:1249345. doi: 10.3389/fped.2023.1249345 (PMC10523391; doi:10.3389/fped.2023.1249345)
Supplement: Supplementary file 1 [file Datasheet1.pdf]

Appendix 1

Description of the Reflective Group Dialogue Intervention following the TIDieR guidelines.

**Items**	**Description**
**1. Brief name**	Reflective Group Dialogue Intervention<br>A systematic method for a healthcare team to observe and reflect on how they communicate and share decision-making with parents during medical rounds.
**2. Why**	Improve parents' active participation in communication and decision-making during medical rounds in NICUs.
**3. What (materials)**	A video-recorded lecture with handouts<br>A guide for conducting the intervention. Includes parent interview questions and a reflective group discussion structure (Appendices 2 and 3).
**4. What (procedures):**	**The intervention consists of theoretical and experiential parts:**<br>**Theoretical part:**<br>The intervention began with a theoretical lecture on the evidence behind the involvement of parents in the medical rounds. (40 min)<br>Practical planning of observation and reflection sessions in the unit. Informing the parents and agreeing on the time of the interview with them. (Approximately one and a half hours in total.)<br>**Experiential part:**<br>1) Observation of a medical round with a patient and her/his parents (20 min)<br>2) Interview with parents about their experience (15 min) (15min)<br>3) Reflective discussion with the healthcare team included in the round and the feedback from the parents' interview (45min)<br><br>**Participants in an observation and reflection session were:**<br>A doctor who was rounding<br>A rounding nurse, the infant's primary nurse, or a nurse who cared for the infant that day<br>An assistant nurse who took care of the infant on that shift<br>Researcher(s)

Appendix 1

Description of the Reflective Group Dialogue Intervention following the TIDieR guidelines.

	The researchers were responsible for 1) documentation of observations during the round, and 2) interviewing parent/s and leading the reflective discussion after the medical round.
**5. Who provided**	**In this study, the following professionals delivered the theoretical teaching, observations, and reflective group discussions:**<br>1. An associated professor of nursing science<br>2. A Ph.D. in health sciences<br>3. A clinical psychologist and PhD<br>4. A psychology student
**6. How**	**Theoretical teaching was** provided:<br>• as a recorded video lecture and handouts<br>**Observation of the medical round and interview of the parents:**<br>• in the unit at bed-side<br>**The reflective discussion** was done:<br>• in groups with the whole participating healthcare team<br>• in a room close to the unit and reserved for the purpose
**7. Where**	The intervention was carried out at the regional level III NICU in Uppsala. A detailed description is available in the method section.
**8. When and how much**	The unit healthcare professionals took part in the intervention from 11/2018 to 12/2018.<br>1. During this 2-month period 9 observation and reflection sessions were carried out in the unit.<br>2. Five unit doctors were included in the intervention. One doctor participated in two observation-reflection sessions. The other 4 participated in one observation-reflection session.<br>3. From 12 NICU nurses and assistant nurses participated in the sessions<br>4. One of the unit's healthcare professionals received training to carry on the observation and reflection sessions independently.
**9. Tailoring**	No tailoring was done during the pilot.
**10. Modifications**	During the implementation, the structure of the reflective group discussion was modified.
**11. How well (planned)**	The fidelity was followed with logbooks on intervention delivery. The reflective discussions were audio recorded and adherence to the reflective discussion structure can be analyzed from the recordings.

Appendix 1

Description of the Reflective Group Dialogue Intervention following the TIDieR guidelines.

	During the implementation, fidelity, and adherence were monitored by comparing the documentation and intervention implementation plan at the end of each case.
**12. How well (actual)**	The fidelity of the theoretical part was not achieved because no one of the participating healthcare team members had reviewed the recorded lecture.<br><br>The other parts of the intervention were delivered as planned.
