# Supplementary material for: Improving NICU staff decision-making with parents in medical rounds: a pilot study of reflective group dialogue intervention
Source: Front Pediatr. 2023 Sep 12;11:1249345. doi: 10.3389/fped.2023.1249345 (PMC10523391; doi:10.3389/fped.2023.1249345)
Supplement: Supplementary file 2 [file Datasheet2.pdf]

Appendix 2
Structure of a reflective group dialogue after observing a medical round with a family

Introduction

We are here to discuss your thoughts and observations about the medical round without judgment. The focus is on parent participation in discussion and decision-making. We are not looking for right or wrong ways of doing things. The reflective discussion is not about judging the round or your behavior during it. Rather, it is about exploring and creating a new understanding of what happened and why in the interaction between you and the parent(s). First, reflection is about exploring what happened and then trying to understand the motivations, experiences, and emotions behind the behavior. Second, it is about playing with optional scenarios of how things might have gone if someone had behaved or said something differently. We kindly ask that you keep this discussion confidential.

Feelings and the most important or confusing moments during the medical round

- How did you find the observation?
- Give positive feedback to the participating healthcare team (*This is a comment to the facilitator*).
- What was the most important event for you during today's round?
- Did you find anything unclear or confusing during the round?
- Feedback from parents' interview; maybe the parent's experience of the most important or unclear moments during the round.

The facilitator of the reflective discussion summarizes all the situations or issues raised by the participants and facilitates exploration and reflection on the communication in the situations or issues one by one. Perhaps starting with the one most often mentioned by the participants or by the parents.

When communication between the staff and parents is explored

Describe how the communication went during the round.

- **Explorative questions:**
  - Did everything go as planned, or was there anything you would have done differently?
  - How did your communication, your colleagues, or the parents' communication contribute to the round?
  - What was your role in the round? How did it affect your communication/behavior?
  - What kind of feelings did you have during the round? How did it affect your communication/behavior?
  - Feedback from the interview with the parent(s).

Reflective questions (Can be used anytime):
  - What if...
  - Can you imagine that…
  - How would that kind of new way to e.g. behave affect you?
  - How would it affect your feelings or thinking?
  - How would it affect parents - their behavior or feelings?
  - How would it affect the parents' feelings or thinking?

When the topics of parents' experiences and participation are explored

- Describe how the parents were involved in the communication or decision-making during the round.

Appendix 2

Structure of a reflective group dialogue after observing a medical round with a family

- **Exploratory questions:**
  - What do you think supported the parents' participation?
  - What did you or your colleagues do to support parents' participation?
  - Were there any other factors that influenced the parents' participation?
  - How do you think that the parents experienced the round? (How do you know this?)
  - Feedback from the interview with the parent(s).
    - Were their experiences similar or different from yours?
  - Reflective questions as above.

When the topics of decision-making are explored or facilitate

- What decisions were made during the meeting?

- **Exploratory questions:**
  - Who made these decisions? How were the decisions made?
  - What role did you, your colleagues, or the parents play in today's decisions?
  - Were parents involved in the decision?
  - What were the consequences of the decision?
  - What feelings did you/your colleagues/parents have during the decision-making process? Or afterward?
  - Feedback from the interview with the parent(s).
    - The parents' experience of the decisions made during the round.
  - Reflective questions as above.

Closing the Reflective Discussion:

- What do you want to take with you from this discussion for future rounds? (e.g., new ideas to support parental involvement, to do as it was done but more consciously because it worked well…)
- Now that you have gone through this, would you like to have similar reflective discussions in the future?
