# Supplementary material for: Improving NICU staff decision-making with parents in medical rounds: a pilot study of reflective group dialogue intervention
Source: Front Pediatr. 2023 Sep 12;11:1249345. doi: 10.3389/fped.2023.1249345 (PMC10523391; doi:10.3389/fped.2023.1249345)
Supplement: Supplementary file 3 [file Datasheet3.pdf]

Appendix 3

Questions to ask parents after the medical round

Questions about the medical round

Start by telling the parents that we want to understand "How you as parents felt the discussion was during today's round?"

• How did you experience today's medical round?

• What are your thoughts and feelings after today's round?

• What made you feel involved in the round?

• What do you remember as the most important event during today's round?

• Did you experience anything during the round as confusing/unclear?

• What decisions were made in the round today? Did you feel that you were involved in the decisions that were made? Did you/you feel that you/you were listened to?

• Do you have anything else you would like to tell us about that day's round?

• Later today we will discuss today's round with the staff who were present. What can we share with them from this discussion?

Questions about the study

• How did you/you feel that researchers observed the round? Did it matter to you that there were more people with you?

• How does it feel to give feedback about the round?

• How would you like to participate in a discussion about the round together with the staff?

• What significance does it have for you personally and for you as a parent to be part of the round?
